# Exploring the Glycosylation of Serum CA125

**DOI:** 10.3390/ijms140815636

**Published:** 2013-07-26

**Authors:** Radka Saldova, Weston B. Struwe, Kieran Wynne, Giuliano Elia, Michael J. Duffy, Pauline M. Rudd

**Affiliations:** 1 NIBRT GlycoScience Group, National Institute for Bioprocessing Research and Training, Fosters Avenue, Mount Merrion, Blackrock, Dublin 4, Ireland; E-Mails: radka.fahey@nibrt.ie (R.S.); weston.struwe@chem.ox.ac.uk (W.B.S.); 2 The Mass Spectrometry Resource, Conway Institute, University College Dublin, Dublin 4, Ireland; E-Mails: kieran.wynne@ucd.ie (K.W.); giuliano.elia@philochem.ch (G.E.); 3 UCD School of Medicine and Medical Science, Conway Institute of Biomolecular and Biomedical Research, University College Dublin, Dublin 4, Ireland; E-Mail: michael.j.duffy@ucd.ie; 4 Department of Pathology and Laboratory Medicine, St Vincent’s University Hospital, Dublin 4, Ireland

**Keywords:** CA125, *N*-glycosylation, biomarker, ovarian cancer

## Abstract

Ovarian cancer is the most lethal gynaecologic cancer affecting women. The most widely used biomarker for ovarian cancer, CA125, lacks sensitivity and specificity. Here, we explored differences in glycosylation of CA125 between serum from patients with ovarian cancer and healthy controls. We found differences between CA125 *N*-glycans from patient sera compared to controls. These include increases in core-fucosylated bi-antennary monosialylated glycans, as well as decreases in mostly bisecting bi-antennary and non-fucosylated glycans in patients compared to controls. Measurement of the glycosylated state of CA125 may therefore provide a more specific biomarker for patients with ovarian cancer.

## 1. Introduction

Ovarian cancer is the most lethal gynaecological malignancy in women [1]. More than 70% of patients are diagnosed at an advanced stage as the disease lacks specific symptoms in early stages [1]. The 5-year survival rate for women diagnosed at the late stage is less than 20%, whereas it is 90% if detected in the early stage [1]. Therefore, early detection may result in better outcomes. CA125 is the most widely used serum marker in the detection and management of the disease [1]. Normal CA125 concentrations are below 30 U/mL [2]. CA125 levels are increased in 80%–85% of women in the advanced stages of ovarian cancer, as opposed to 50% of women in stage I of ovarian cancer. Therefore, CA125 is useful for monitoring progression and regression, rather than for early diagnosis [1]. Elevated levels of CA125 have also been found elevated in benign conditions such as endometriosis, pregnancy, ovulatory cycles, liver diseases, congestive heart failure, and in infectious disease such as tuberculosis [2]. CA125 is also one of several mucin cancer biomarkers. Other mucins, such as MUC1 (breast cancer) or prostate specific antigen (PSA, prostate cancer), have been used for cancer detection and progression monitoring, however, their sensitivity and specificity are limited. The quantities of these biomarkers in serum are low, as they are shed from tissue into the bloodstream [3,4].

The quantification of soluble CA125 levels is currently performed with a second generation of assays. These assays are based on double determinant ELISA tests that use two monoclonal antibodies (mAb) directed against the M11 and OC125 epitope groups [2]. Anti-CA125 antibodies are divided into three groups, OC125-like (group A), M11-like (group B), and Ov197-like which each recognize domains of non-overlapping epitopes [2]. All three types of antibodies can recognize either native or denatured CA125. OC125 antibody exhibits a reduced binding after treatment with Peptide -*N*-Glycosidase F (PNGase F) and currently available anti-CA125 antibodies do not permit fine discrimination among various CA125 species [2].

**Figure 1:**
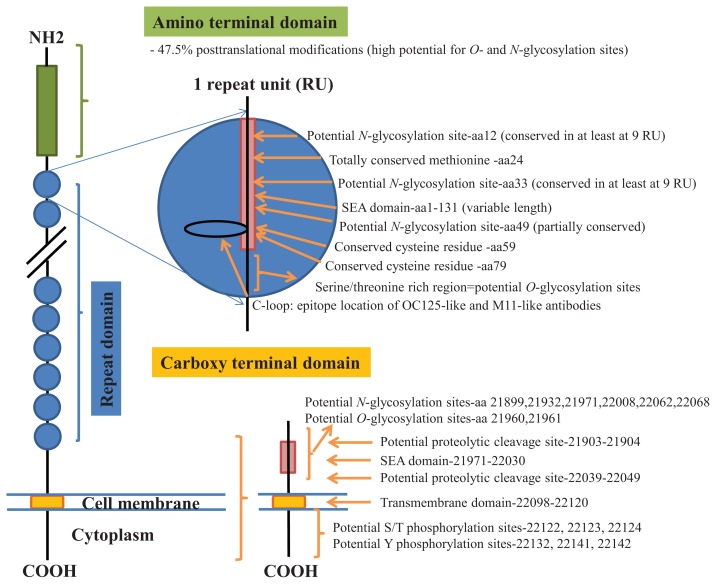
Schematic model of CA125. A schematic overview of the molecular structure of CA125 as modified from Weiland *et al.* [10]. CA125 has 249 potential *N*-glycosylation sites (UniProt, http://www.uniprot.org/uniprot/Q8WXI7) and over 3700 *O*-glycosylation sites (whole sequence was submitted to ISOGlyP, http://isoglyp.utep.edu/index.php, using early ppGalNAc transferases-T1, T2, T5 and T12). A typical repeat unit is pictured in detail as well as the carboxy terminal domain. The total length of the protein is 22,152 amino acids.

CA125 antigen was first identified in six epithelial ovarian carcinoma cell lines and tumour tissue of ovarian cancer patients reacting with monoclonal antibody OC 125 [5]. CA125 is a 200–2000 kDa mucin-type molecule (MUC16 mucin) with abundant *N*- and *O*-glycans (249 potential *N*-glycosylation and over 3700 *O*-glycosylation sites, Figure 1). The carbohydrate content was previously estimated to be 24%–28% [6,7]. It is a large transmembrane glycoprotein (Figure 1) [8]. The CA125 protein core is composed of a short cytoplasmic tail, a transmembrane domain and a large glycosylated extracellular structure [8]. The extracellular domain is characterized by a large number of tandem repeats of SEA domains (sea-urchin sperm protein, enterokinase and agrin) which encompass an interactive disulfide bridged cysteine-loop and the site of OC125 and M11 binding [2,8]. The molecule also includes an amino terminal domain of serine/threonine-rich sequences which may account for most of the *O*-glycosylation known to be present in CA125 [8]. Release or secretion of CA125 appears to be directly linked to the epithelial growth factor receptor signal transduction pathway [2]. A functional role of CA125 in the physiological context or cancer remains unknown, however several properties of CA125 that may be of relevance for its biological function have been proposed. CA125 has been suggested to play a role as a lubricant, preventing adhesion of membranes [7]. The glycan structures that are present on CA125 have been implicated in immune suppression, raising the possibility that CA125 might help protect the embryo from maternal immune rejection and play an immunoevasive role in ovarian cancer [7,9].

CA125 binds to galectin-1 and mesothelin [2,11]. CA125 from human peritoneal fluid was shown to enhance the invasiveness of a benign endometriotic cell line, which raises the possibility that CA125 plays a role in endometriosis [12]. Several other mucins have been implicated in invasion and metastasis of cancer, partly because of similar functions. For example, MUC1 induces T cell apoptosis and increases invasiveness, MUC18 has been implicated in tumour angiogenesis, MUC2 enhances colon cancer metastasis to the liver, although it appears to inhibit initial neoplasia, MUC8 is up-regulated in metastatic medulloblastoma, and MUC3B is up-regulated in intestinal metaplasia [7]. Previous studies investigated the glycosylation of CA125 from OVCAR3 cell lines, amniotic fluid and placenta, but not from serum. Wong *et al.* analysed the major *N*-glycans and *O*-glycans of CA125 from the OVCAR3 cell line by mass spectrometry [9]. In this study, 20% of the *N*-glycans were found to be of the high mannose type and 80% of the complex type structures [9]. Complex glycans were mostly mono-fucosylated bi-antennary, tri-antennary and tetra-antennary bisected structures with not more than one sialic acid [9]. *O*-glycans were both core 1 and 2 type glycans with branched core 1 antennae.

Milutinovic *et al.* analysed CA125 glycans from amniotic fluid with lectins [13]. VVA (Vicia villosa agglutinin, specific for terminal GalNAc) and SBA (Glycine max agglutinin, specific for GalNAcα1-Ser/Thr and/or GalNAcGalβ1,3GalNAcα1-Ser/Thr) showed the strongest binding [13]. WGA (wheat germ agglutinin, specific for GlcNAc and its β1,4 oligomers) also reacted strongly, and LCA (Lens culinaris agglutinin, specific for Fucα1,6Man) and UEA (Ulex europaeus agglutinin, specific for Fucα1,2) showed lower reactivity [13]. H2 antibody (recognising fucose α1,2 bound to terminal Gal epitopes) showed no reaction [13]. The strong reaction of VVA and SBA indicates the predominance of *O*-glycans and that the glycans are both core- and outer arm-fucosylated [13]. Jankovic *et al.* analysed CA125 from placenta with lectins [14]. CA125 bound most strongly to WGA and RCA (Ricinus communis agglutinin, specific for terminal Gal linked β1,4 to GlcNAc), but low affinity interactions occurred with the other lectins such as ConA (Canavalia ensiformis, specific for high mannose glycans), SNA (Sambucus nigra agglutinin, specific for sialic acid linked α2,6 to Gal), MAA (Maackia amurensis agglutinin, specific for sialic acid linked α2,3 to Gal), AAA (Aleuria aurantia agglutinin, specific for core-fucosylated *N*-glycans, outer arm-fucosylated glycans are retarded), SBA and PNA (Arachis hypogaea agglutinin, specific for Galβ1,3 GalNAcα1-Ser/Thr) [14]. Placental CA125 showed both core- and outer arm-fucosylated, mono- and di-sialylated glycans linked both α2,3 and α2,6 [14]. WGA and RCA reactivity indicated the presence of polylactosamine structures or the presence of *O*-glycans. Based on PNA and SBA binding, these *O*-glycans are Galβ1–3GalNAc and (Galβ1–3GlcNAcβ1–6) GalNAc [14].

In our previous glycosylation studies on breast cancer MUC1, pancreatic cancer RNAse1 and prostate cancer PSA, we found different glycosylation in tumour origins [3,15,16]. In this study we attempted to investigate the glycosylation of CA125 as a possible improved serum biomarker for the detection of ovarian cancer. We used controls with CA125 values above normal 30 U/mL to evaluate significance of our results.

## 2. Results

**Figure 2:**
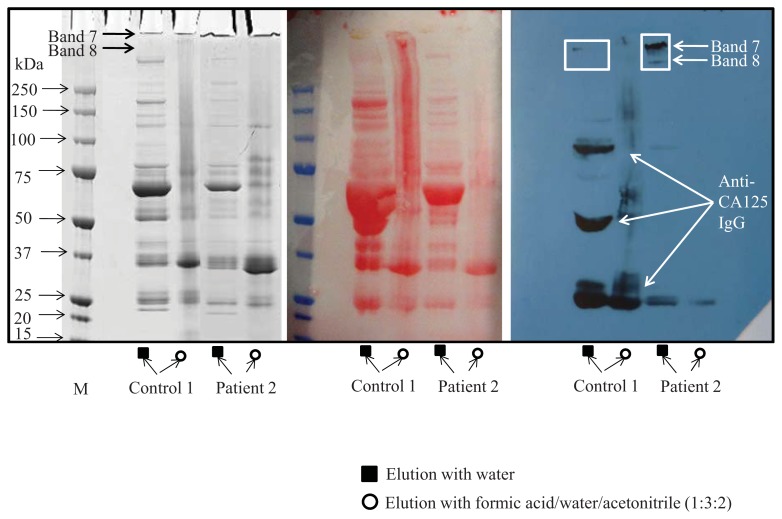
SDS-PAGE and Western blot identification of CA125. Elution of CA125 from the immunoaffinity column was performed with water and with formic acid/acetonitrile (1:3:2). Approximately 300U (1.2 μg) of purified CA125 was run on two SDS-PAGE gels, one was stained with Coomassie blue, and the proteins of the other gel were transferred to a PVDF membrane. Proteins were visualized with Ponceau staining in order to check the complete transfer from the gel to the membrane. Western blot analysis with anti-CA125 antibody was performed. CA125 is present bands 7 and 8, both on the Coomassie stained gel and the Western blot (anti-CA125 antibody is cross reacting with secondary antibody rabbit anti-mouse).

The isolation of CA125 by immunoadsorption was optimized according to Peter *et al.* [17]. Elution with water as well as formic acid/water/acetonitrile (1:3:2) was tested. The presence of CA125 was identified by Western blot in the water eluate in bands 7 and 8 (Figure 2). Amount of CA125 isolated was analysed by CA125 ELISA. CA125 from all patients and age-matched controls was isolated using this method and bands 7 and 8 were cut from the SDS-PAGE gel and used for glycan analysis.

CA125 was also identified with 1 unique peptide by LC-MS/MS (found peptide sequence was blasted against Uniprot Swissprot databases and was identified as human CA125. CA125 is heavily *N-*and *O*-glycosylated (Figure 1), so the protein is not easily accessible to trypsin due to steric hindrance caused by the glycan structures. In all preparations, also other glycoproteins were identified, however, their protein scores were lower and their relative percentage in most samples was below 3%.

### *N*-Glycosylation of CA125 Reveals Differences between Healthy Controls and Patients with Ovarian Cancer

**Figure 3:**
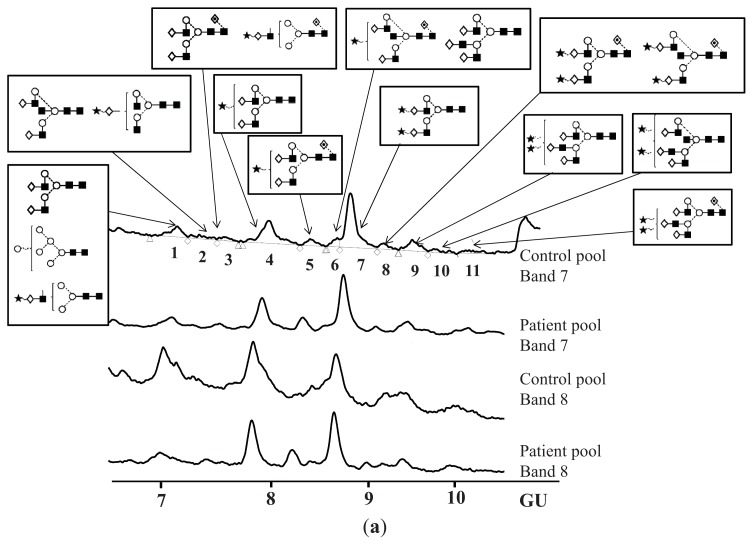

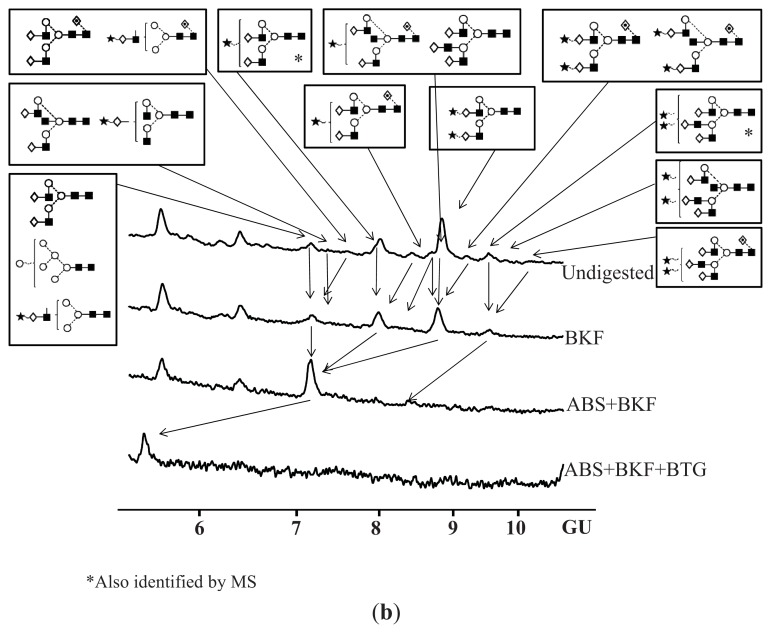
CA125 *N*-glycans from pooled controls and patients and their assignments. (**a**) HILIC-HPLC chromatograms of bands 7 and 8 from pooled controls and patients including structures assigned in each peak; (**b**) Assignments of CA125 *N*-glycans on pooled samples-example control pool band 7, undigested HILIC-HPLC profile and digestions with BKF, ABS + BKF, ABS + BKF + BTG.

**Figure 4:**
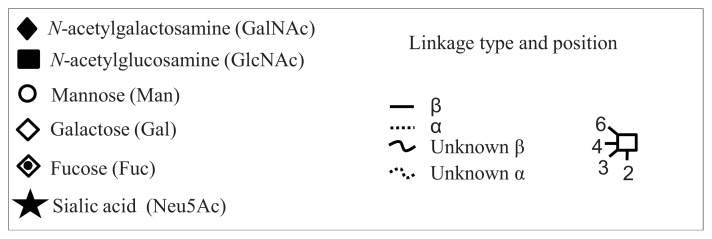
Structural symbols for the glycans, their linkages and abbreviations used.

As low quantities of *N*-glycans were obtained from individual samples, pooled samples were analysed for more accurate assignments (Table 1, Figure 3). The *N*-glycans of CA125 from human serum contained mono-, bi- and tri-antennary glycans, mostly mono- and di-sialylated, many of them were also core-fucosylated and bisected, and some were high-mannosylated (Table 1, Figure 4).

The differences between pooled controls and patients are shown in Table 2. Core-fucosylated bi-antennary monosialylated glycans were increased and mostly bisecting bi-antennary and non-fucosylated glycans were decreased in patients compared to controls.

**Figure 5:**
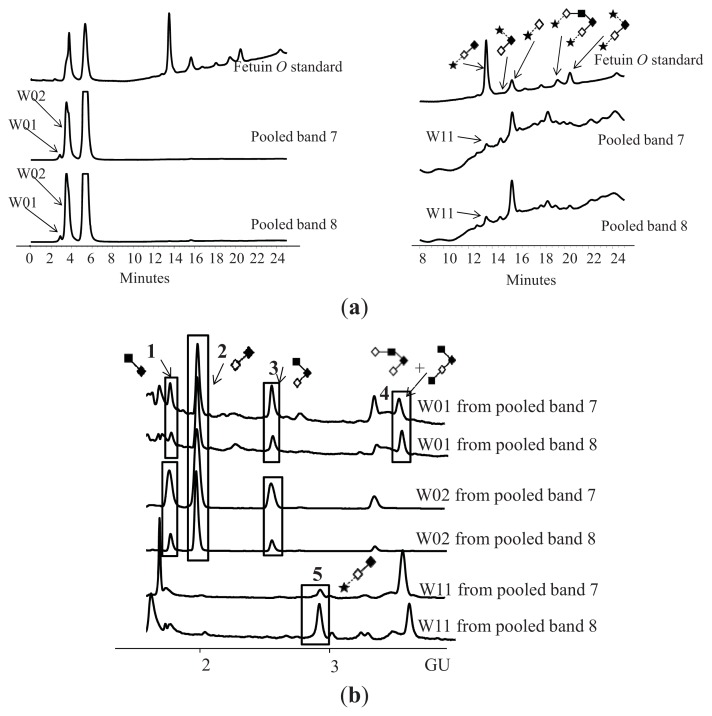

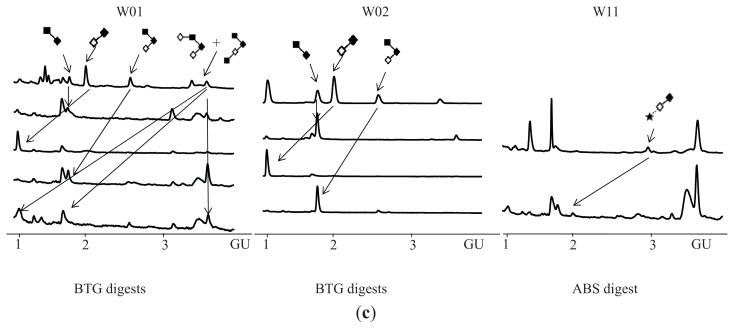
CA125 *O*-glycans from pooled patients and their assignments. (**a**) WAX-HPLC profiles of pooled bands 7 and 8 with fractions highlighted; (**b**) HILIC-HPLC profiles of WAX fractions with assigned glycans highlighted; (**c)** Assignments of CA125 *O*-glycans on pooled samples-example pooled band 7, fractions W01 and W02 were digested with BTG and fraction W11 was digested with ABS.

As low quantities of *O*-glycans were obtained from individual samples, pooled patient serum was analysed for more accurate assignments and quantifications (Figure 5). The *O*-glycans of CA125 obtained from human serum contained core 1 structures (GlcNAcβ1–6GalNAc, Galβ1–3GalNAc = T-antigen) and core 2 structures (Galβ1–3[GlcNAcβ1–6]GalNAc, Galβ1–4GlcNAcβ1–6[Galβ1–3]GalNAc, GlcNAcβ1–6[GlcNAcβ1–3Galβ1–3]GalNAc, NeuNAca2–3Galβ1–3GalNAc) (Table 3). No significant differences between controls and patients were found.

## 3. Discussion

As different glycosylation was previously found on breast cancer MUC1, pancreatic RNAse1 and prostate cancer PSA [3,15,16], we attempted to look at the glycosylation status of CA125. Glycosylation of CA125 from the ovarian cancer cell line OVCAR3, amniotic fluid and placenta was previously reported [9,13]. However, glycosylation of CA125 from serum would be more convenient for a potential biomarker. This is the first report of CA125 glycosylation in serum and comparison of CA125 from ovarian cancer to controls.

### Glycosylation of CA125 from Serum Reveals Differences in *N*-glycans between Controls and Ovarian Cancer Patients

In order to isolate CA125, large quantities of serum were required to obtain a minimum of 300 U (1 μg). Despite this, only low amounts of glycans were purified. Also, small quantities of glycoproteins co-purified with CA125, however, in contrast to all proteins, CA125 has a much higher abundance of *N*- and *O*-glycosylation sites (Figure 1). Our reported glycosylation from serum CA125 was different than the glycosylation previously published on OVCAR3 cell lines, amniotic fluid and placenta [9,13,14].

We found complex type *N*-glycans which were mono-, bi- and tri-antennary, mostly mono- and di-sialylated, many of them were core-fucosylated, some bisected, and also the presence of high-mannosylated *N*-glycans. Ovarian cancer patients showed increases in core-fucosylated bi-antennary monosialylated glycans and decreases in mostly bisecting bi-antennary and non-fucosylated glycans compared to healthy controls. The *O*-glycans consisted of core 1 (GlcNAcβ1–6GalNAc, Galβ1–3GalNAc = T-antigen) and core 2 structures (Galβ1–3[GlcNAcβ1–6]GalNAc, Galβ1–4GlcNAcβ1–6[Galβ1–3]GalNAc, GlcNAcβ1–6[GlcNAcβ1–3Galβ1–3]GalNAc, NeuNAca2–3Galβ1–3GalNAc). The relative percentage of *N*-glycans of CA125 from OVCAR3 cells were 20% high mannose type and 80% complex type structures [9]. They were mostly mono-fucosylated bi-antennary, tri-antennary and tetra-antennary bisected structures with no more than one sialic acid [9]. The *O*-glycans of CA125 from OVCAR3 cells were both core 1 and 2 type glycans with branched core 1 antennae [9]. The glycans from amniotic fluid showed the predominance of *O*-glycans and were both core- and outer arm-fucosylated [13]. Placental CA125 showed both core- and outer arm-fucosylated, mono- and di-sialylated glycans linked both α2,3 and α2,6 and lower amounts of high-mannosylated glycans [14]. *O*-glycans were also detected and the structures determined were Galβ1–3GalNAc and (Galβ1–3GlcNAcβ1–6) GalNAc [14].

The differences in glycosylation of CA125 from various sources could reflect the source of the protein. This has been noted before, for example in the cases of cell line specific glycosylation of PSA [16,20] and RNAse I [15,21]. Also, glycosylation is tissue specific, which suggests alternate roles or locations of the protein within the same organism [22]. Therefore it is not surprising that CA125 from serum, amniotic fluid, placenta and OVCAR3 cell lines have specific glycosylation patterns.

Wong *et al.* analysed the major *N*-glycans and *O*-glycans of CA125 on OVCAR3 cell line by mass spectrometry [9]. Milutinovic *et al.* and Jankovic *et al.* analysed CA125 glycans from amniotic fluid and placenta using lectins [14]. High/ultra high performance liquid chromatography (HPLC/UPLC), mass spectrometry (MS) and lectin analysis are the most common glyco-analytical techniques used by glycobiologists in the characterization of glycan structures [23]. UPLC is a very sensitive technique, and labelling glycans with a fluorophore enables released glycans to be detected at the femtomole level [23]. Glycans are labelled stoichiometrically (1:1) in a structurally unbiased manner with 2-aminobenzamide (2-AB), allowing accurate relative quantitative measurements and comparison between samples [18]. Lectins are easy to use, however, they can have broad specificity and the level of detection (100 picomole levels) is not as low as HPLC or MS [24].

The most common glycosylation changes in cancer are increases in size, branching and sialylation of the *N*-glycans [25]. The most common terminal glycan epitopes found on glycoproteins on the surface of cancer cells are; sialyl Lewis x (sLex), sialyl Lewis a (Lea), sialyl Tn, Globo H, Lewis y and polysialic acid [25]. SLex was found on acute phase proteins and is also increased during inflammation [25]. In ovarian cancer, there is also an increase in agalactosylated glycans and a shift from alpha2–3 to alpha2–6 linked sialic acid [26]. *O*-glycans from cancer cells are usually shorter and more sialylated than those from normal cells [27]. This is due to the fact that the increase of sialyltransferase activity prevents further *O*-glycan processing, and thus terminates *O*-glycan chains, due to the blocking effect of sialic acid on GlcNAc- or galactosyl-transferases [27]. Therefore in cell types with high sialyltransferase activity such as tumour cells, produced *O*-glycans are short and sialylated [27]. Core 2 *O*-glycans allow tumour cells to evade natural killer cells of the immune system and survive longer in the circulatory system, thereby promoting tumour metastasis [28]. We found mostly core 2 structures, but not many sialylated glycans on CA125 which could explain the role of CA125 in promoting cancer metastasis.

## 4. Experimental Section

### 4.1. Serum Samples

Patient serum samples were obtained from St. Vincent’s University Hospital Dublin. These patients had CA125 requested and all had advanced ovarian cancer. All were Caucasian between 45 and 70 years of age, their CA125 levels were 215.8–1977 U/mL. Following measurement of CA125, all samples were anonymised and used for glycan analysis. Control sera obtained from Innovative research (Patricell Ltd) were from 58 and 61 years old white females, their CA125 levels were 63.2 U/mL and 39.8 U/mL. Samples were collected with informed consent from healthy donors. 450 mL of whole blood was collected on a cold pack, centrifuged at 5000× *g* for 10 min. After centrifugation, the serum was transferred from the red blood cells to a blood transfer bag. The serum was stored at room temperature for 24–48 h until it had finished clotting. After the serum had clotted, it was centrifuged ant 5000× *g* for 15 min. The serum was transferred to a sterile plastic bottle and stored at −20 °C.

### 4.2. CA125 Isolation from Serum Samples

Method of immunoadsorption according Peter *et al.* for PSA [17] was optimized for CA125 isolation. Four milligrams of streptavidin coated magnetic beads (Roche, 10 mg/mL) were washed three times with 400 μL of washing buffer (10 mM TRIS, 150 mM NaCl, pH 7.5, 0.5% Tween 20) using magnetic separation. Meantime, 60 μg biotinylated mouse monoclonal antibody anti-CA125 (Hytest, MAb X306, 2.9 mg/mL) was prepared in 400 μL of incubation buffer (10 mM TRIS, 150 mM NaCl, pH 7.5, 0.1% Tween-20, 1% BSA) and added to the washed beads. The mixture was incubated for 30 min at room temperature (RT) under gentle shaking. The beads were then washed three times with 400 μL of washing buffer. 400 μL of serum was prepared (containing approximately 300 U = 1.2 μg of CA125) and added to the washed beads with bound anti-CA125 antibody. The mixture was incubated for 1 h, at RT, slightly shaking. The beads were then washed three times with 400 μL of washing buffer. CA125 was eluted with 250 μL of water for 1 h at RT, slightly shaking and the beads were washed with 50 μL of water; and then eluted with 250 μL of formic acid/water/acetonitrile (1:3:2) for 1 h, RT, slightly shaking and the beads were washed with 50 μL of formic acid/water/acetonitrile (1:3:2) and added to the eluate.

### 4.3. SDS-PAGE Electrophoresis

Electrophoresis in 4%–12% Bis-Tris SDS-PAGE mini-gels (Invitrogen, Carlsbad, CA, USA) was performed at room temperature according to the method of Laemmli [29]. The gels were Coomassie and Ponceau stained. All samples were reduced with 5% 2-mercaptoethanol before analysis.

### 4.4. Western Blot

Isolated proteins from samples were after electrophoresis transferred to a PVDF membranes (400 mA constant for 2 h) and Ponceau stained. Membranes were blocked with Tris-buffered saline Tween (TBST, 10 mM TRIS, 100 mM NaCl, pH 7.5, 0.2% Tween 20) with 5% dry milk for 1 h at RT. Membranes were washed three times 10 min with TBST before an overnight incubation at 4 °C in 2.9 μg/mL anti-CA125 (Hytest, MAb X306) in the TBST-1% dry milk. Membranes were washed three times 10 min with TBST before 1.5 h incubation with 0.04 μg/mL secondary rabbit anti-mouse (Abcam, Ab6728). The blots were developed using the ECL Plus chemiluminescent detection system (GE Healthcare, Uppsala, Sweden).

### 4.5. Release and Purification of *N*- and *O*-Glycans from Human Serum CA125 in Gel Block

*N*-glycans were released from serum CA125 by *in situ* digestion with Peptide -*N*-Glycosidase F (PNGase F, Roche, Mannheim, Germany) in SDS-PAGE gel bands as described earlier [28]. Briefly, isolated CA125 gel bands obtained from SDS-PAGE were alkylated, washed and *N*-glycans released by PNGase F. *O*-glycans were released from SDS-gel blocks after *N*-glycans release using ammonia-based β-elimination [30].

### 4.6. Fluorescent Labelling of the Reducing Terminus of *N*-Glycans

Glycans were fluorescently labelled with 2-aminobenzamide (2AB) by reductive amination [31] (LudgerTag 2-AB labelling kit LudgerLtd., Abingdon, UK).

### 4.7. Exoglycosidase Digestion of 2AB Labelled *N*-Linked Glycans

All enzymes were purchased from Prozyme, San Leandro, CA, USA. The 2AB-labelled glycans were digested in a volume of 10 μL for 18 h at 37 °C in 50 mM sodium acetate buffer, pH 5.5, using arrays of the following enzymes: ABS—*Arthrobacter ureafaciens* sialidase (EC 3.2.1.18), NAN1—*Streptococcus pneumoniae* sialidase (EC 3.2.1.18), 1 U/mL; BTG—bovine testes β-galactosidase (EC 3.2.1.23), 1 U/mL; BKF—bovine kidney alpha-fucosidase (EC 3.2.1.51), 1 U/mL; GUH—β-N-acetylglucosaminidase cloned from *Streptococcus pneumonia*, expressed in *E. coli* (EC 3.2.1.30), 4 U/mL.

After incubation, enzymes were removed by filtration through a protein binding EZ filters (Millipore Corporation, Beford, MA, USA) [32], the *N*-glycans were then analysed by HILIC.

### 4.8. Hydrophilic Interaction Liquid Chromatography (HILIC)

HILIC was performed using a TSK-Gel Amide-80 4.6 × 250 mm column (Anachem, Luton, UK) on a 2695 Alliance separations module (Waters, Milford, MA, USA) equipped with a Waters temperature control module and a Waters 2475 fluorescence detector. Solvent A was 50 mM formic acid adjusted to pH 4.4 with ammonia solution. Solvent B was acetonitrile. The column temperature was set to 30 °C. The 3 h gradient started with a linear gradient of 20%A and went up continuously over 152 min to 58%A at a flow rate of 0.4 mL/min. Samples were injected in 80% acetonitrile [18]. Fluorescence was measured at 420 nm with excitation at 330 nm. The system was calibrated using an external standard of hydrolyzed and 2AB-labelled glucose oligomers to create a dextran ladder, as described previously [32]. Experimentally determined reproducibility for the quantitation of the HILIC peaks was found to be 2%–30% (in average 9%) relative standard deviation using ten individually prepared and analysed aliquots of serum from the same sample (Saldova *et al.*, in preparation).

### 4.9. Weak Anion Exchange Chromatography (WAX)—High Performance Liquid Chromatography (HPLC)

WAX-HPLC was performed using a Vydac 301VHP575 7.5 × 50-mm column (Anachem) on a 2695 Alliance separations module with a 474 fluorescence detector (Waters). Solvent A was 0.5 M formic acid adjusted to pH 9.0 with ammonia solution, and solvent B was 10% (*v*/*v*) methanol in water. Gradient conditions were as follows: a linear gradient of 0 to 5% A over 12 min at a flow rate of 1 mL/min, followed by 5% to 21% A over 13 min and then 21% to 50% A over 25 min, 80% to 100% A over 5 min, and then 5 min at 100% A. Samples were injected in water. A fetuin *N*-and *O*-glycan standards were used for calibration [32,33].

### 4.10. Negative Ion Electrospray Ionisation Mass Spectrometry ESI-MS and ESI MS/MS

Samples were analysed by static nanoelectrospray ionization using a Waters (Waters MS Technologies, Manchester UK) tandem quadrupole time-of-flight mass spectrometer. Samples were diluted with a 1:1 (*v:v*) mixture of water:methanol containing 0.1 mM ammonium phosphate. MS and MS/MS data was acquired in negative mode with the following instrument settings: source temperature 120 °C, capillary voltage 1.3 kV, cone voltage 100 V and the RF-1 voltage was 130 V. MS/MS precursor ions were selected with a 3 m/z mass window and were fragmented by CID using argon as the collision gas. CID voltage was altered accordingly from 20 to 40 V. Data acquisition and data processing were conducted with Waters MassLynx version 4.1. Interpretation of the negative ion MS/MS spectra was according to published work [34–37].

### 4.11. Protein Identification by Mass Spectrometry (MS)

Samples were run on a Thermo Scientific LTQ ORBITRAP XL mass spectrometer connected to an Exigent NANO LC.1DPLUS chromatography system. Tryptic peptides were resuspended in 0.1% formic acid. Each sample was loaded onto a Biobasic C18 PicofritTM column (100 mm length, 75 mm ID) and was separated by an increasing acetonitrile gradient, using a 60 min reversed phase gradient (7%–40% acetonitrile for 40 min) at a flow rate of 300 nL/min. The mass spectrometer was operated in positive ion mode with a capillary temperature of 200 °C, a capillary voltage of 46 V, a tube lens voltage of 140 V and with a potential of 1900 V applied to the frit. All data was acquired with the mass spectrometer operating in automatic data data-dependent switching mode. A high resolution MS scan was performed using the Orbitrap to select the 5 most intense ions prior to MS/MS analysis using the Ion trap. The precursor accurate mass was <20 ppm and the MS/MS fragment mass tolerance was + or −0.8 Da.

The raw data was analysed using Bioworks Browser 3.3.1 SP1, a proteomics analysis platform. All MS/MS spectra were sequence database searched using the algorithim TurboSEQUEST. The MS/MS spectra were searched against a non-redundant human Swissprot database. The following search parameters were used: precursor-ion mass tolerance of 20 ppm, fragment ion tolerance of 1.0 Da with methionine oxidation and cysteine carboxyamidomethylation specified as differential modifications and a maximum of 2 missed cleavage sites allowed. Each peptide used for protein identification met specific parameters, *i.e.*, XCorr values of ≥1.9, ≥2.5, ≥3.2 for single-, double-, and triple- charged peptides, respectively, and a peptide probability of <0.001.

### 4.12. CA125 ELISA

CA125 levels after immunoadsorption were measured using Cancer antigen CA125 enzyme immunoassay test kit (BC-1013, BioCheck, Foster City, CA, USA) according to manufacturer’s instructions.

## 5. Conclusions

We found that CA125 from ovarian cancer patient sera have increased core-fucosylated bi-antennary monosialylated glycans, compared to controls and have decreased mostly bisecting bi-antennary and non-fucosylated glycans. We described important differences between the serum CA125 and published CA125 from OVCAR3 cells, amniotic fluid and placenta. Specific glycosylation patterns on a protein marker rather than the protein levels, as currently measured, could be used as more specific and sensitive biomarkers. In the case of PSA for the detection of prostate cancer, several studies have already shown that glycosylation improves the diagnosis of prostate cancer [4]. Combination of the current biomarkers with the glyco-biomarkers is a potential source of further increases in the sensitivity and specificity of diagnosis tools, particularly for early tumour detection [38]. For example, altered glycosylation could be presented in the early stages of lung cancer [39], where some of the glycosylation changes (highly sialylated, branched and outer arm fucosylated glycans) were detectable as early as in stage I. Monitoring core-fucosylation levels in alpha-fetoprotein is a sensitive marker for early tumour detection [40]. In early stage breast cancer patients, combined levels of core-fucosylated agalactosylated bi-antennary glycans and glycans containing sLex were significantly increased in lymph node positive patients [41]. Also, recently, microarray glycoprofiling of CA125 was shown to improve ovarian cancer diagnosis [42]. Collectively, these findings suggest that measurement of the glycosylated state of CA125 may provide a more specific biomarker for patients with ovarian cancer. However, to access the utility of CA125 glycosylation as a potential biomarker in clinics, further experiments will be needed to verify more samples using high-throughput methods.

## Supplementary Information

Figure S1 The major *N*-glycans from CA125 were detected as singly and doubly charged ions. CID of the A2G2S1 (*m*/*z* 1930 M-H-) and A3G3S2 (*m*/*z* 1293 M-2H2-) ions from CA125. Negative ion fragments confirmed the presence of the most abundant structures identified by HPLC. Fragment ion nomenclature was as proposed by Domon and Costello [43].

## Figures and Tables

**Table 1:** Serum CA125 glycans.

Peak number	GU	1 Abbreviation	2 Structures
1	7.08	M6	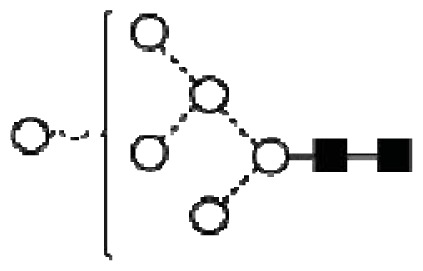
		A2G2	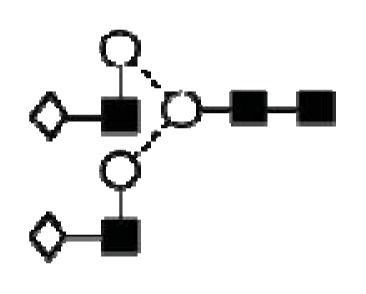
		A1G1S1	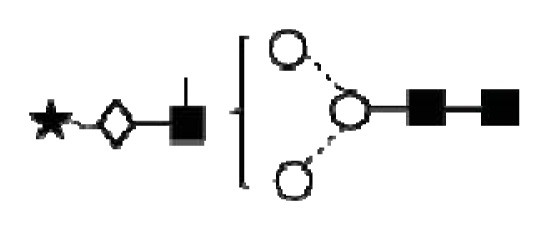
2	7.23	A2BG2	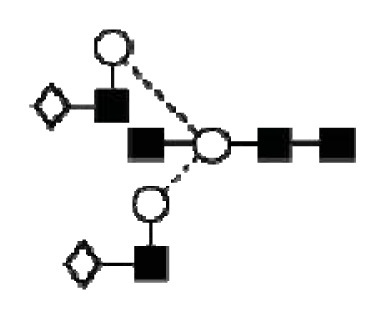
		A2G1S1	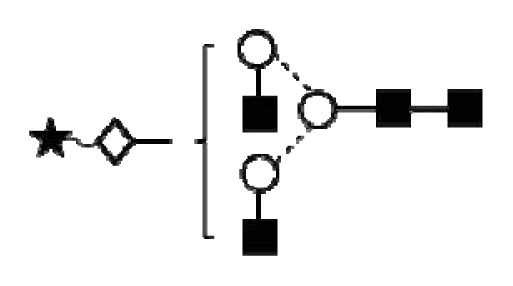
3	7.47	FA2G2	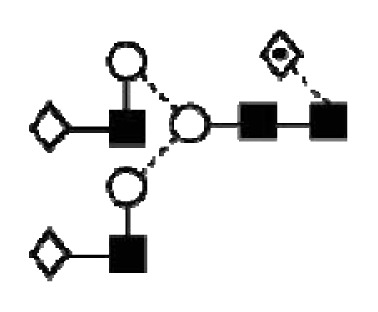
		FA1G1S1	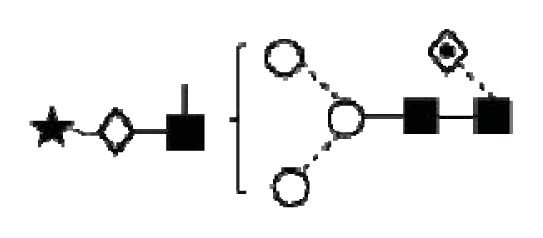
4	7.92	3A2G2S1	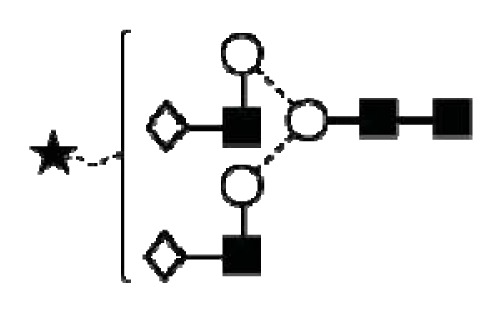
5	8.34	FA2G2S1	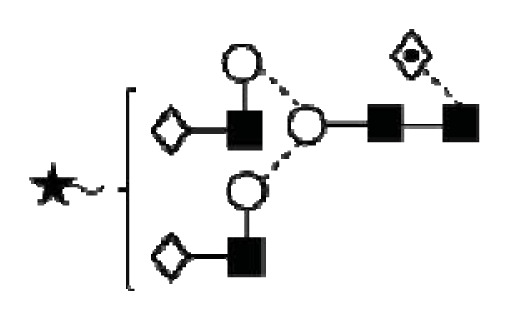
6	8.55	FA2BG2S1	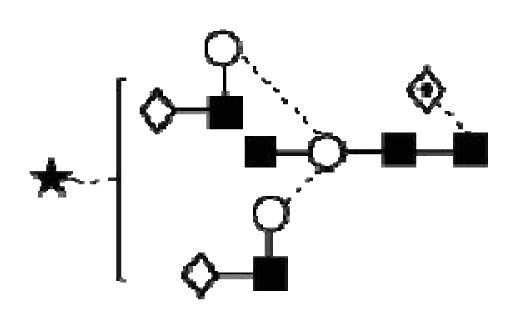
		A3G3	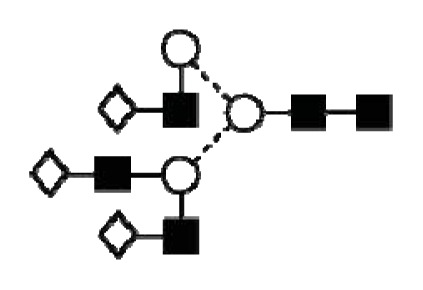
7	8.75	A2G2S2	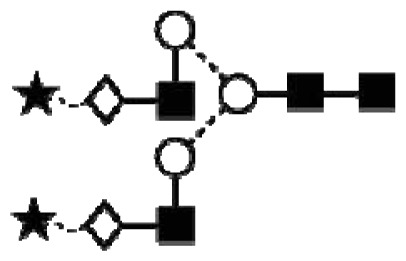
8	9.10	FA2G2S2	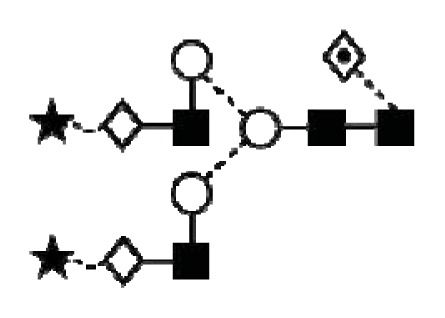
		FA2BG2S2	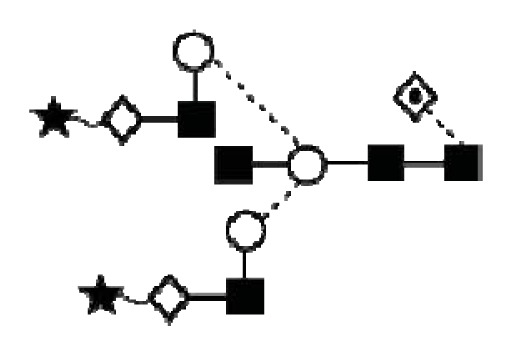
9	9.37	3A3G3S2	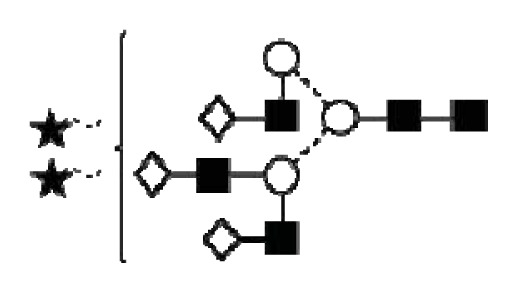
10	9.58	A3BG3S2	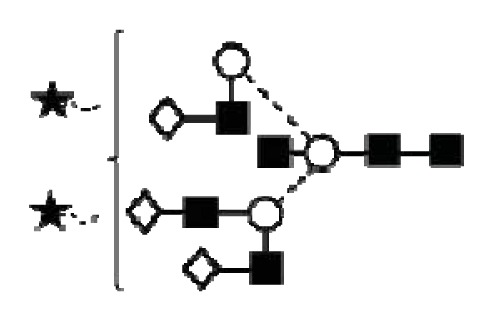
11	10.11	FA3G3S2	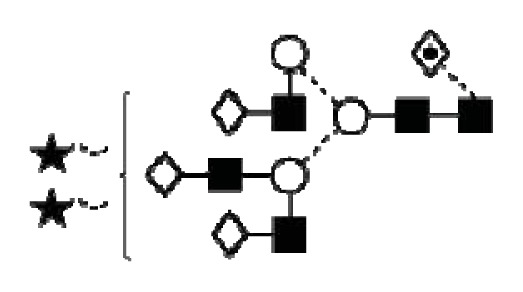

1 Structures are abbreviated in accordance to previous publications by Royle *et al.* [18], Harvey *et al.* [19] and as used in GlycoBase 3.2 (http://glycobase.nibrt.ie/database/show_nibrt.action). All *N*-glycans have two core-*N*-Acetylglucosamines (GlcNAc) and a trimannosyl core. F at the start of the abbreviation indicates a core-fucose linked α1–6 to the core GlcNAc; Aa, represents the number a of antenna (GlcNAc) on the trimannosyl core linked to the mannose arm; B, bisecting GlcNAc linked β1–4 to β1–4 core-mannose; Gc, represents the number c of galactose linked β1–4 on antenna and Sd, represents number d of sialic acids linked to the galactose.

2 The graphical symbols used to represent the different sugar residues and linkages are shown in Figure 4.

3 Analysis of CA125 *N*-glycans by mass spectrometry was performed by direct nano-infusion where two abundant structures (A2G2S1 and A3G3S2) were detected. Fragmentation of these precursor ions yielded MS/MS spectra that allowed for confirmation of these structures (as seen in the included Figure S1).

**Table 2:** Differences in *N*-glycosylation between individual controls and patients.

Band	7 Glycan(s)	Controls	Patients	Fold difference
		% Area 1		
Peak 2	A2BG2+A2G1S1	4.08 ± 0.57	2.16 ± 1.09	1.9
Peak 5	FA2G2S1	10.02 ± 3.78	18.64 ± 10.40	1.9
Peak 6	FA2BG2S1+A3G3	10.02 ± 0.07	4.10 ± 2.03	2.4
**Band 8**	**Glycan(s)**	**Controls**	**Patients**	**Fold difference**
		**% Area**^1^		
Peak 1	A2G2+M6+A1G1S1	19.87 ± 6.24	4.90 ± 4.11	4.1
Peak 2	A2BG2+A2G1S1	10.28 ± 2.75	6.06 ± 3.68	1.7
Peak 5	FA2G2S1	3.15 ± 2.18	8.96 ± 1.30	2.8

1 Based on 2 controls and 7 patients (band 7) or 5 patients (band 8), peaks with significant change in peak areas in patients compared to controls are highlighted (green-decreased and red-increased).

**Table 3:** Serum CA125 *O*-glycans.

Peak number	GU	Abbreviation	Structures 1	
1	1.72	GlcNAcß1–6GalNAc	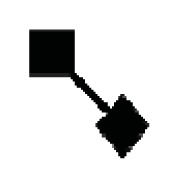	Core 1
2	1.96	Galß1–3GalNAc	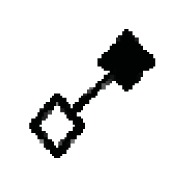	Core 1
3	2.59	Galß1–3[GlcNAcß1–6]GalNAc	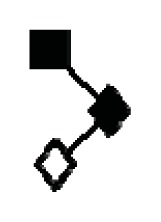	Core 2
4	3.60	Galß1–4GlcNAcß1–6[Galß1–3]GalNAc	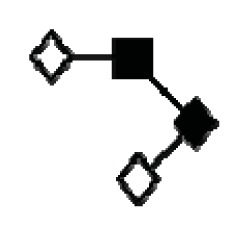	Core 2
		GlcNAcß1–6[GlcNAcß1–3Galß1–3]GalNAc	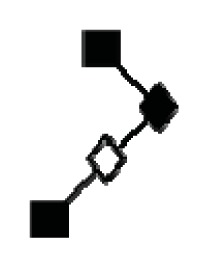	Core 2
5	2.94	NeuNAca2–3Galß1–3GalNAc	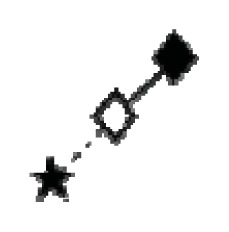	Core 2

1 The graphical symbols used to represent the different sugar residues and linkages are shown in Figure 4.
