# Supplementary figures and images for: Exploring the Glycosylation of Serum CA125
Source: Int J Mol Sci. 2013 Jul 26;14(8):15636–54. doi: 10.3390/ijms140815636 (PMC3759877; doi:10.3390/ijms140815636)

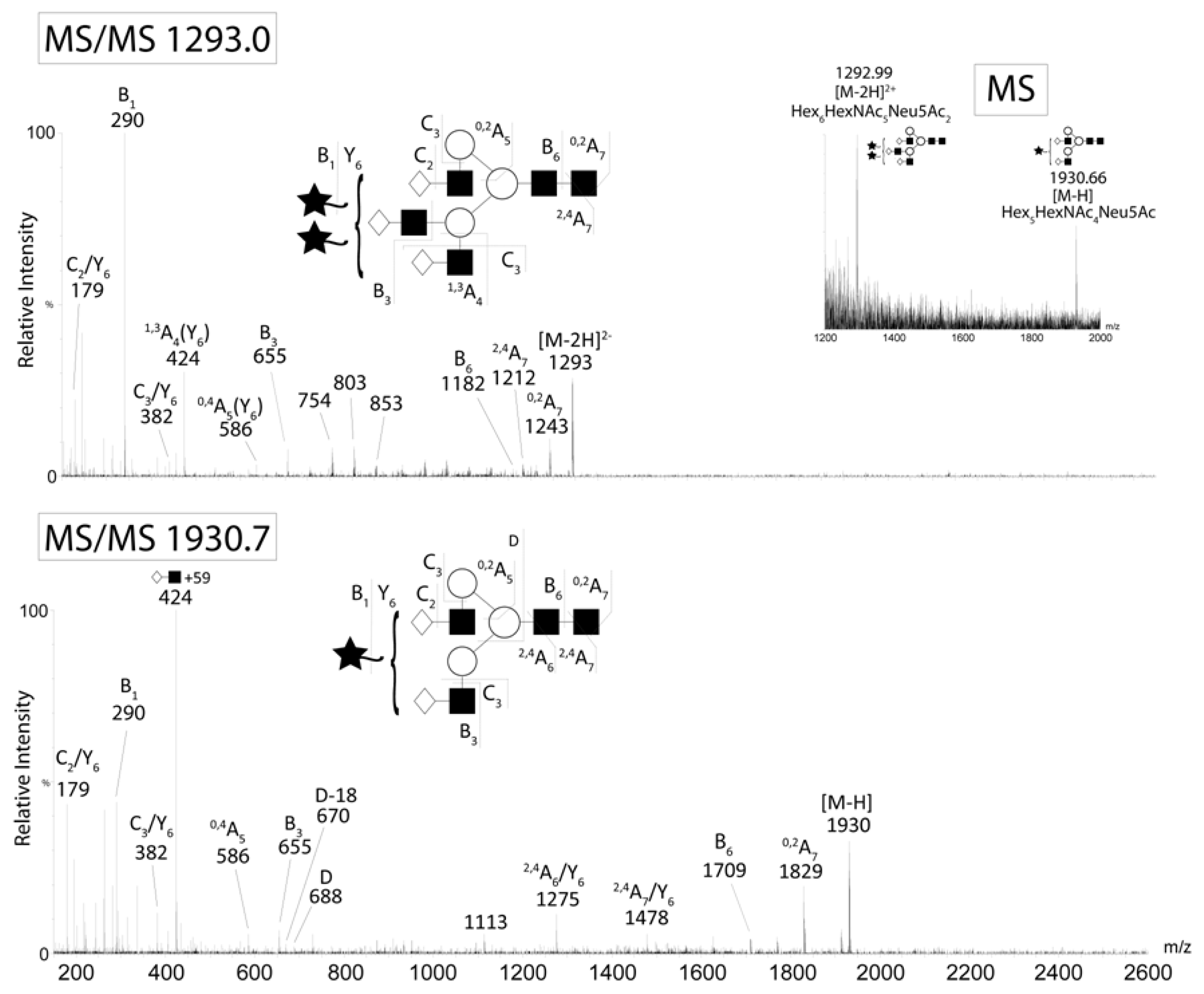

Supplement: Figure S1 — The major N-glycans from CA125 were detected as singly and doubly charged ions. CID of the A2G2S1 (m/z 1930 M-H-) and A3G3S2 (m/z 1293 M-2H2-) ions from CA125. Negative ion fragments confirmed the presence of the most abundant structures identified by HPLC. Fragment ion nomenclature was as proposed by Domon and Costello [43]. [file ijms-14-15636s1.tif]
